# Antisense Oligonucleotide-mediated Suppression of Muscle Glycogen Synthase 1 Synthesis as an Approach for Substrate Reduction Therapy of Pompe Disease

**DOI:** 10.1038/mtna.2014.57

**Published:** 2014-10-28

**Authors:** Nicholas P Clayton, Carol A Nelson, Timothy Weeden, Kristin M Taylor, Rodney J Moreland, Ronald K Scheule, Lucy Phillips, Andrew J Leger, Seng H Cheng, Bruce M Wentworth

**Affiliations:** 1 Genzyme, A Sanofi Company, Framingham, Massachusetts, USA; 2 Translational Medicine Consulting, Westford, Massachusetts, USA

**Keywords:** antisense oligonucleotide, cell penetrating peptide, glycogen synthase, morpholino, nonsense-mediated decay, Pompe disease, phosphorodiamidate morpholino oligonucleotide, skeletal muscle, substrate reduction therapy

## Abstract

Pompe disease is an autosomal recessive disorder caused by a deficiency of acid α-glucosidase (GAA; EC 3.2.1.20) and the resultant progressive lysosomal accumulation of glycogen in skeletal and cardiac muscles. Enzyme replacement therapy using recombinant human GAA (rhGAA) has proven beneficial in addressing several aspects of the disease such as cardiomyopathy and aberrant motor function. However, residual muscle weakness, hearing loss, and the risks of arrhythmias and osteopenia persist despite enzyme therapy. Here, we evaluated the relative merits of substrate reduction therapy (by inhibiting glycogen synthesis) as a potential adjuvant strategy. A phosphorodiamidate morpholino oligonucleotide (PMO) designed to invoke exon skipping and premature stop codon usage in the transcript for muscle specific glycogen synthase (Gys1) was identified and conjugated to a cell penetrating peptide (GS-PPMO) to facilitate PMO delivery to muscle. GS-PPMO systemic administration to Pompe mice led to a dose-dependent decrease in glycogen synthase transcripts in the quadriceps, and the diaphragm but not the liver. An mRNA response in the heart was seen only at the higher dose tested. Associated with these decreases in transcript levels were correspondingly lower tissue levels of muscle specific glycogen synthase and activity. Importantly, these reductions resulted in significant decreases in the aberrant accumulation of lysosomal glycogen in the quadriceps, diaphragm, and heart of Pompe mice. Treatment was without any overt toxicity, supporting the notion that substrate reduction by GS-PPMO-mediated inhibition of muscle specific glycogen synthase represents a viable therapeutic strategy for Pompe disease after further development.

## Introduction

Acid α-glucosidase (GAA) is a lysosomal enzyme that catalyzes the breakdown of glycogen to glucose. Mutations in the GAA gene that lead to a reduction in the amount or activity of the enzyme are the molecular basis of Pompe disease (glycogen storage disease type II). This autosomal recessive metabolic myopathy results as a consequence of the progressive accumulation of undegraded glycogen, primarily in the lysosomes of cardiac and skeletal muscle. Patients with Pompe disease (incidence of ~1 in 40,000) present with a broad spectrum of disease severity that is inversely correlated with the amount of residual enzyme activity. Complete loss of enzyme activity results in an infantile presentation (so called “floppy babies”) with affected individuals rarely living beyond 2 years of age. Varying degrees of residual enzyme activity lead to a progressive myopathy in young adults as well as older individuals that is invariably fatal.^1^

Pompe disease is managed by periodic infusions of recombinant human GAA (rhGAA) that gained regulatory approval in 2006.^2^ In clinical trials, the infusion of rhGAA improves cardiomyopathy, walking ability, prolongs survival,^3,4,5,6,7,8,9^ and stabilizes pulmonary function in patients.^10^ However, patients treated with enzyme therapy still present with some aspects of the disease.^10^ For example, residual muscle weakness and hearing loss are still evident, and the risk for developing arrhythmias, dysphagia, and osteopenia remains undiminished.^11^ These residual deficits may be due to inefficient delivery of the enzyme to some tissues or to the host immune response to the administered protein. Modified forms of rhGAA conjugated with mannose 6-phosphate-bearing oligosaccharides^12^ or engineered to express a portion of IGF-1^13^ have been developed with improved delivery to muscle and enhanced bioactivity in animal studies. In addition, a small molecule chaperone that improves the stability of the enzyme and enhances glycogen clearance in Pompe mice is being tested clinically.^14^ Gene therapy with recombinant AAV vectors encoding GAA is also being evaluated.^15,16^

Substrate reduction that abates the production of glycogen represents another therapeutic strategy for lysosomal storage disorders. The merits of this concept have been demonstrated for Gaucher and Fabry disease.^17,18,19^ Cytoplasmic glycogen polymers are synthesized by glycogen synthase, whose activity is suppressed by phosphorylation of serines 641 and 645 in a process controlled by the mTORC1 pathway.^20^ Glycogen synthase enzyme activity in Pompe mice is greatly elevated and this increased activity is suppressed by rapamycin treatment leading to lower glycogen levels in skeletal muscle.^21^ Treating Pompe mice with rapamycin effectively reduced glycogen buildup in skeletal muscle, and when used in combination with rhGAA infusions lowered glycogen levels in skeletal muscle and diaphragm more effectively than either agent alone. Rapamycin treatment did not affect glycogen clearance in the heart, an organ already well served by rhGAA, perhaps due to the relatively high levels of the cation-independent mannose 6-phosphate receptors in cardiac tissue. An advantage of rapamycin as a substrate reduction approach was that its impact on glycogen synthase was restricted to muscle, with no effect on the liver enzyme isoform. However, a disadvantage was that the lowest dose of rapamycin effective at reducing glycogen accumulation was also immunosuppressive.

We evaluated the knock down of glycogen synthase for reducing muscle glycogen levels in Pompe mice. Skeletal muscle glycogen synthase activity is the result of transcription of the *Gys1* gene (OMIM 606800). In contrast, liver glycogen synthase activity is generated mostly by expression of the *Gys2* gene (OMIM 138571) and its encoded enzyme produces glycogen as a ready store of glucose for body-wide metabolism. Recent progress in developing therapies for Duchene muscular dystrophy has demonstrated that it is possible to deliver a therapeutically relevant dose of a phosphorodiamidate morpholino oligonucleotide (PMO) for the purpose of skipping mutant exons and generating a truncated, albeit functional dystrophin.^22^ The PMO dose needed to restore dystrophin synthesis can be greatly reduced if it is conjugated to a cell penetrating peptide (PPMO).^23^ We have employed this strategy to induce exon skipping of the Gys1 mRNA for the purpose of reducing its transcript levels, presumably via nonsense mediated decay, with concomitant reductions in the skeletal muscle enzyme. Treating Pompe mice with a PPMO targeting a specific sequence in exon 6 of Gys1 mRNA (GS-PPMO) led to a dose-dependent reduction of the Gys1 transcript in skeletal muscle and heart but not the Gys2 transcript in liver. Likewise, the glycogen synthase protein level was reduced in skeletal muscle and heart and glycogen synthase activity was restored to that of control mice. Consequently, glycogen accumulation was completely abated in skeletal muscle; the impact was less in the heart. We believe these results indicate that substrate reduction by ASO-mediated knock down of skeletal muscle glycogen synthase represents a plausible adjuvant therapy for Pompe disease.

## Results

### A PMO confers selective knockdown of Gys1 mRNA in murine muscle

We wanted to selectively reduce the expression of the isoform of glycogen synthase found mainly in skeletal muscle and heart and so we designed a collection of PMOs using published methods^28^ with the potential to induce exon skipping in the cognate Gys1 but not the Gys2 transcript. Putative exon skipping PMOs were designed to provoke use of out-of-frame premature stop codons existing in the Gys1 transcript to effect the production of an unstable mRNA prone to nonsense-mediated decay. Incorporation of a nonsense codon would also be expected, after translation, to lead to a nonfunctional enzyme.

Candidate PMOs were first tested by direct injection into the TA muscle of mice followed by electroporation. Two weeks later, Gys1 mRNA levels were quantified. Twelve PMOs were tested and two resulted in what appeared to be a substantial reduction in Gys1 mRNA (**Table 1**). One PMO sequence in particular (number 10 in **Table 1**) targeted the skipping of exon 6 and was evaluated further.

**Figure 1:**
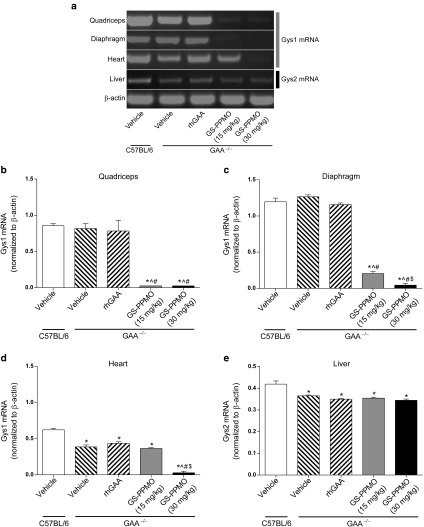
**Gys1 mRNA levels are reduced in skeletal and cardiac muscles of Pompe mice treated with repeated intravenous injections of GS-PPMO.** (**a**) Gel electrophoresis of semi-quantitative polymerase chain reaction (PCR) products derived from samples of RNA isolated from tissues of wild-type and Pompe mice that received the indicated treatments to determine Gys1 transcript levels. Gys1 mRNA levels (normalized to β-actin mRNA levels) were quantified in the (**b**) quadriceps, (**c**) diaphragm, and (**d**) heart tissues. (**e**) Liver was examined for the impact of GS-PPMO on Gys2 mRNA levels. Data represent mean ± SEM, *n* = 9–10 mice per group as indicated in the Materials and Methods. *P* < 0.05, (*) compared to WT, (^) compared to vehicle, (#) compared to rhGAA, ($) compared to GS-PPMO at 15 mg/kg.

The selected PMO was synthesized with a 5′ primary amine to facilitate conjugation of a well-characterized arginine-rich cell penetrating sequence that had been shown previously to facilitate muscle delivery.^29^ This conjugated PMO (GS-PPMO) was injected through a tail vein into Pompe mice once every two weeks for a total of 12 weeks. Age-matched Pompe mice administered saline vehicle or 20 mg/kg recombinant α-glucosidase (rhGAA) on the same schedule served as treatment controls. Age-matched wild-type (C57Bl/6) mice served as untreated controls. Gel electrophoresis analysis of RT-PCR products derived from tissue extracts from Pompe mice at the end of the study showed that treatment with either 15 or 30 mg/kg GS-PPMO significantly reduced Gys1 mRNA levels in the quadriceps and diaphragm muscle (**Figure 1a**–**c**). Gys1 mRNA levels were also dramatically reduced in the heart, but only after treatment with the higher dose (**Figure 1d**). No significant changes were seen in the steady-state levels of Gys2 mRNA in the liver, indicating that GS-PPMO-mediated knockdown was specific for the muscle isoform of glycogen synthase (**Figure 1e**). As expected, treating animals with rhGAA had no impact on Gys1 mRNA levels in skeletal muscle or heart nor the Gys2 mRNA levels in liver.

### Systemic administration of GS-PPMO to Pompe mice selectively reduces glycogen synthase 1 levels

**Figure 2:**
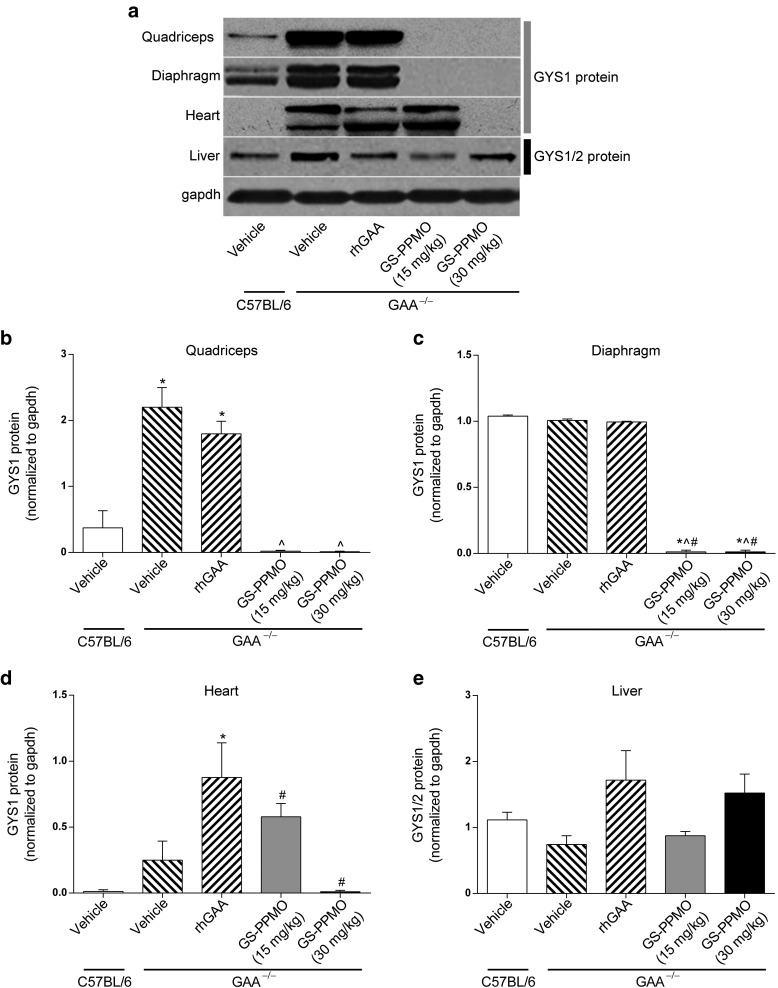
**Gys1 protein levels are reduced in skeletal and cardiac muscles of Pompe mice treated with repeated intravenous injections of GS-PPMO.** (**a**) Western blot analysis was carried out on samples of protein lysates to assess GYS1 protein levels in tissues of wild-type and Pompe mice that received the indicated treatments. GYS1 protein levels (normalized to gapdh protein levels) were quantified in the (**b**) quadriceps, (**c**) diaphragm, and (**d**) heart tissues. (**e**) Liver was examined for the impact of GS-PPMO on Gys1/2 protein levels. Data represent mean ± SEM, *n* = 9–10 mice per group as indicated in the Materials and Methods. *P* < 0.05, (*) compared to WT, (^) compared to vehicle, (#) compared to rhGAA, ($) compared to GS-PPMO at 15 mg/kg.

Western blots were used to assess the degree to which the reductions in Gys1 mRNA led to concomitant reductions in glycogen synthase protein in muscle and liver. In Pompe mice, glycogen synthase protein was found to be substantially elevated in the skeletal muscle and in the heart compared to C57Bl/6 control animals (**Figure 2a**–**e**). This increase was not due to higher levels of mRNA (**Figure 1**) and may therefore be related to an increase in the stability of the enzyme in these Pompe tissues.^21^ However, there were no differences in the levels of glycogen synthase in the liver of Pompe mice compared to those of wild-type control animals.

Treatment of Pompe mice with GS-PPMO (15 or 30 mg/kg) lowered the amount of glycogen synthase in the quadriceps and diaphragm to wild-type levels (**Figure 2a**–**c**). Treatment also reduced the elevated levels of glycogen synthase in the Pompe mouse heart, and achieved complete correction at the higher (30 mg/kg) dose (**Figure 2d**). Neither dose of GS-PPMO affected the amount of total glycogen synthase in the liver (**Figure 2e**). Treating Pompe mice with rhGAA also did not significantly alter the amount of glycogen synthase in the tissues tested.

### Systemic administration of GS-PPMO reduces glycogen synthase activity in the skeletal muscle and heart of Pompe mice

**Figure 3:**
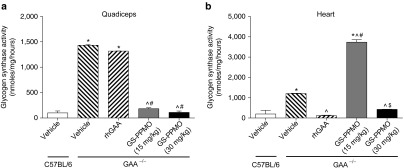
**Glycogen synthase activity is decreased in the quadriceps and heart muscles of Pompe mice treated with GS-PPMO.** Glycogen synthase activity in the (**a**) quadriceps and (**b**) heart of wild-type and Pompe mice was assayed as described in the Materials and Methods. Data represent mean ± SEM, *n* = 9–10 mice per group as indicated in the Materials and Methods. *P* < 0.05, (*) compared to WT, (^) compared to vehicle, (#) compared to rhGAA, ($) compared to GS-PPMO at 15 mg/kg.

Treating Pompe mice with GS-PPMO led to complete correction of the elevated glycogen synthase activity in the quadriceps (**Figure 3a**). A reduction of enzyme activity in the heart was observed only in Pompe mice treated at the higher dose of 30 mg/kg GS-PPMO (**Figure 3b**), whereas an apparent increase in activity was noted at the lower dose. In general, these findings are consistent with the mRNA and protein measurements noted above. Treating Pompe mice with rhGAA (at the dose tested) had no effect on glycogen synthase activity in the skeletal muscle but lowered that in the heart to normal levels. This differential response to treatment with the recombinant enzyme is consistent with previous reports in Pompe mice.^12^

### Systemic treatment with GS-PPMO abates accumulation of tissue glycogen in Pompe mice

**Figure 4:**
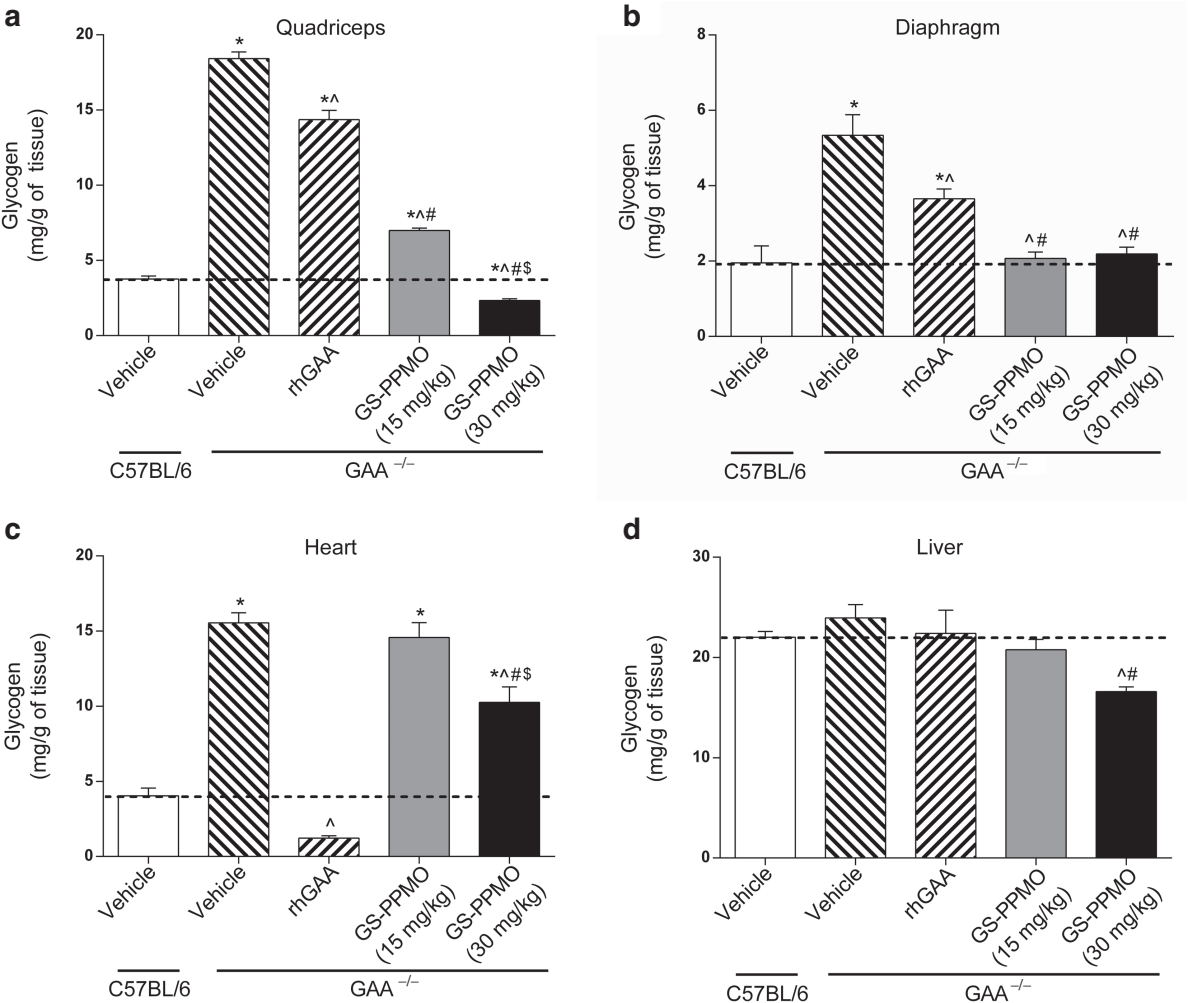
**Accumulation of lysosomal glycogen is abated in skeletal muscle of Pompe mice treated with GS-PPMO.** Glycogen levels in the (**a**) quadriceps, (**b**) diaphragm, (**c**) heart, and (**d**) liver of Pompe and wild-type mice were measured using the amplex red assay described in the Materials and Methods. Data represent mean ± SEM, *n* = 9–10 mice per group as indicated in the Materials and Methods. Dashed line represents mean glycogen level of wild-type mice. *P* < 0.05, (*) compared to WT, (^) compared to vehicle, (#) compared to rhGAA, ($) compared to GS-PPMO at 15 mg/kg.

Tissue extracts were subjected to quantitative glycogen analysis to determine whether the noted reductions in glycogen synthase protein levels and activity in GS-PPMO-treated Pompe mice also resulted in a concomitant lowering of lysosomal glycogen accumulation. Treating Pompe mice with GS-PPMO led to a dose-dependent decrease in glycogen accumulation in the quadriceps and heart (**Figure 4a**,**c**), and a reduction to the level found in normal control mice in the diaphragm at both doses tested (**Figure 4b**). In the quadriceps and diaphragm of Pompe mice treated with 30 mg/kg GS-PPMO, the levels of glycogen were reduced to those found in wild-type C57Bl/6 mice. As expected, Pompe mice treated with 20 mg/kg rhGAA showed a partial reduction in glycogen levels in the quadriceps, diaphragm and a greater reduction in the heart (**Figure 4a**–**c**). Neither treatment with rhGAA nor GS-PPMO (15 mg/kg) had an impact on glycogen levels in the liver. However, treatment with the higher dose (30 mg/kg) of GS-PPMO did result in a partial reduction in liver glycogen levels (**Figure 4d**).

### Systemic administration of GS-PPMO does not elicit overt changes in histopathology and blood chemistry

**Figure 5:**
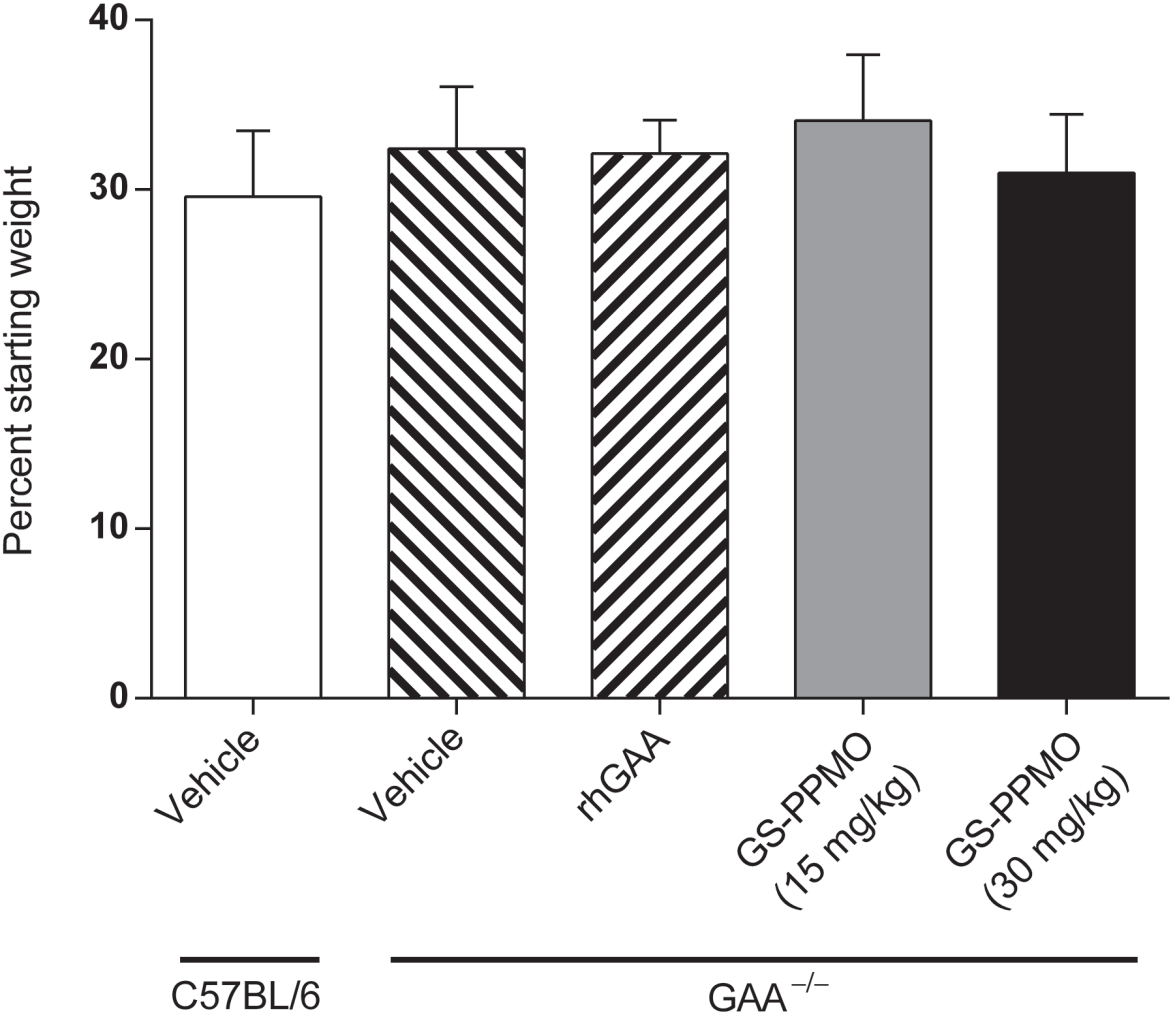
**Body weights of animals measured at the end of the study.** Data are presented as mean ± SEM, *n* = 9–10 mice per group as indicated in the Materials and Methods.

**Figure 6:**
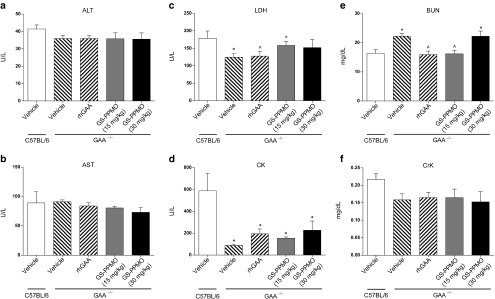
**Serum chemistries of Pompe mice treated with GS-PPMO compared to control animals.** (**a–f**) Levels of ALT, AST, LDH, CK, BUN, or CK in serum collected 24 hours after the final dose. Data represent mean ± SEM, *n* = 9–10 mice per group as indicated in the Materials and Methods. *P* < 0.05, (*) compared to WT, (^) compared to vehicle.

**Figure 7:**
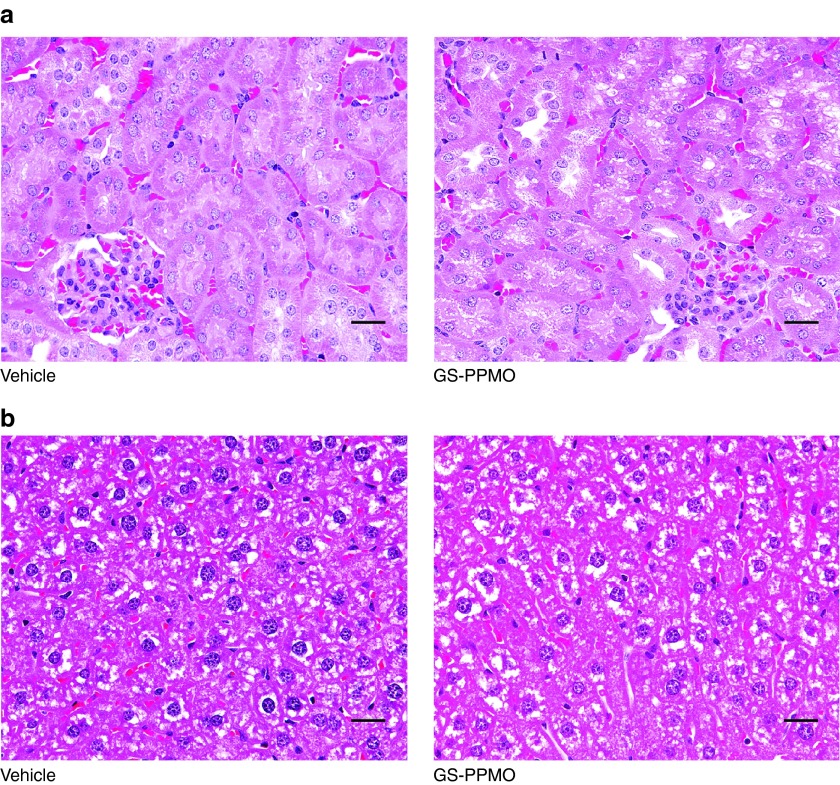
**Histopathological analysis of kidney and liver of Pompe and wild-type mice. Hematoxylin and eosin stained slides were prepared from mice treated with either vehicle or GS-PPMO (30 mg/kg) as indicated.** (**a**) Kidney sections of GS-PPMO-treated Pompe mice show a normal architecture of proximal convoluted tubules and glomeruli. (**b**) Livers of GS-PPMO-treated Pompe mice exhibit the presence of Kupffer cells in hepatocytes. Scale bar = 25 μm.

We also investigated the potential toxicologic impact of GS-PPMO treatment and knockdown of GYS1 mRNA to assess the therapeutic index associated with administering this particular type of antisense oligonucleotide for Pompe disease. Pompe mice treated with either dose of GS-PPMO did not demonstrate significant differences in weight gain from mice in the control cohort (**Figure 5**). Examination of blood biomarkers of liver, muscle, and kidney damage also did not reveal any deviations from those noted in control mice (**Figure 6a**–**f**). Finally, histological analysis of the kidney and liver of GS-PPMO-treated Pompe mice revealed normal architecture and the absence of discernible lesions (**Figure 7**). These data suggest that systemic administration of GS-PPMO is well tolerated, at the doses tested.

## Discussion

Enzyme replacement therapy is effective at addressing several of the disease manifestations noted in both early and late-onset Pompe disease. However, therapeutic penetrance in some patients is suboptimal leading to the desire to identify adjuvant therapies that may improve the quality of care afforded these patients. Modified rhGAA (neoGAA) promises to be a more potent form of enzyme replacement therapy by improving targeting to the CI-M6P receptor.^12^ If successful, an substrate reduction therapy approach could be coupled with rhGAA treatment in Pompe patients with more severe disease to effect more rapid clearance of glycogen from skeletal muscle.

Recent studies employing a double genetic knockout of *Gys1* and *GAA* demonstrate characteristics of glycogen accumulation in a model of Pompe disease, cardiomegaly and type II muscle fiber atrophy, can be abrogated by knocking out *Gys1*.^30^ In the same study, it was shown that *Gys1*/*GAA* double knockouts had better tetanic force generation and fatigue resistance compared to *GAA* knockout animals. The concept of Gys1 protein knockdown has been further examined through the use of siRNA designed to degrade Gys1 mRNA after viral delivery using AAV2/1. Two-day-old Pompe mice injected with virus targeting Gys1 mRNA were partially corrected for the buildup of glycogen measured 4 weeks later.^31^ We have extended the concept of SRT for Pompe disease by developing a systemically-deliverable peptide conjugated morpholino (GS-PPMO) the mechanism of action for which is based on exon skipping. Antisense oligonucleotides, including PMO compounds, are in clinical testing for the induction of exon skipping in subgroups of Duchene muscular dystrophy patients.^22^ In that case, ASOs are designed to force the splicing machinery to bypass a mutation-containing exon in the dystrophin mRNA and join with the next in-frame splice junction to yield a truncated yet functional dystrophin protein. In this study, we have designed and tested a PMO that forces exon skipping in the processing of Gys1 mRNA and results in a shift in reading frame leading to a premature termination codon and degradation of the message—probably by nonsense-mediated decay. We selected a specific morpholino, GS-PMO, for systemic administration based on short-term screening of candidate PMOs for knock down of Gys1 mRNA by direct injection into skeletal muscle. The selected PMO was modified for systemic administration by conjugating a cell penetrating peptide to it (creating GS-PPMO) to improve its biodistribution to muscle.

GS-PPMO was capable of provoking Gys1 mRNA decreases in quadriceps, diaphragm, and heart in a dose-dependent manner. The bioactivity seen in the heart was only significant at the higher dose tested, a finding consistent with PPMOs tested for exon skipping of dystrophin (data not shown). GS-PPMO activity at the mRNA level appeared to be sequence specific as there was no impact on the liver isoform, Gys2. This finding was expected given that GS-PPMO is complementary to intron sequence in *Gys1*. The fact that GS-PPMO appears specific for the muscle enzyme suggests that its action will not interfere with systemic glucose mobilization in Pompe patients, which is governed by the predominant liver enzyme encoded by the *Gys2* gene. Also as expected, administration of rhGAA had no impact on the steady state level of Gys1 or Gys2 mRNA in any tissue tested.

The GS-PPMO-mediated knock down of Gys1 mRNA greatly reduced the amount of Gys1 protein in quadriceps and diaphragm at both doses tested, and also in the heart at the higher dose. These findings are impressive given the elevation of the protein in these tissues seen in Pompe mice compared to control animals. There was no change in the level of liver Gys2 protein that is consistent with the designed specificity of GS-PPMO toward Gys1 mRNA. We further evaluated the effects of GS-PPMO treatment on glycogen synthase enzyme activity in the quadriceps and heart and found substantial reduction in both tissues as well as some noteworthy differences. Glycogen synthase activity was considerably elevated in the quadriceps and heart of Pompe mice; a finding consistent with previous reports.^32^ Treating Pompe mice with GS-PPMO reduced activity in these tissues to very near the wild-type levels found in C57Bl/6 mice at both doses tested. This was also true in the heart but only with the higher dose employed. It is remarkable to note that even with the elevated level of GS-activity in the untreated Pompe heart, the administration of low dose GS-PPMO (15 mg/kg) increased GS-activity further by about threefold. Glycogen synthase activity is regulated at the level of protein phosphorylation that is controlled by environmental conditions through the mTOR pathway. It is not clear how treatment with GS-PPMO at 15 mg/kg elevated GYS1 protein, a property it shared with GAA administration (**Figure 2d**). However, GS-PPMO given at 15 mg/kg also raised glycogen synthase activity in the heart compared to vehicle-treated animals. The effect of elevating glycogen synthase protein and activity above baseline in the heart is completely resolved at the higher dose tested. This effect highlights the challenges that may be encountered in manipulating the glycogen storage system in this disease.

We also assessed the effects of rhGAA and GS-PPMO treatment on glycogen build up in Pompe mice. Analysis of both quadriceps and diaphragm after treatment with rhGAA at 20 mg/kg revealed modest declines in glycogen compared to vehicle-treated Pompe mice. Treatment with GS-PPMO was significantly more effective at abating glycogen build up in the quadriceps and diaphragm, the latter tissue being normalized to wild-type levels at the low dose. A complete abatement of glycogen build up was observed in the hearts of mice treated with rhGAA to levels below those in untreated C57Bl/6 mice. This may be due to the well-noted presence of CI-M6P receptors in the heart permitting greater efficacy of rhGAA in that tissue. GS-PPMO treatment resulted in a modest dose-dependent decline of glycogen build up in heart. While the glycogen reduction is not of the magnitude seen in skeletal muscle it is possible that longer treatment would eventually result in further declines. It is also worth noting that the heart is an organ well served by rhGAA enzyme therapy.

The development of PPMO for human use is significantly challenged by renal toxicity of the drug candidate observed in primate studies.^33^ This toxicity is likely due to the cationic nature of the peptide and is observed at doses required for efficacious exon skipping in the primate musculature. These previous studies were conducted with the same prototypic peptide that was used in the GYS1 studies reported herein. Thus, safer peptide conjugates will need to be developed or the dosing regimen refined before these results can be translated into a clinical therapy. However, the studies reported here demonstrate proof of concept for GYS1 knockdown as a therapeutic approach for Pompe disease if these limitations can be overcome. For example, Pompe patients might be treated effectively with a combination of rhGAA and a PPMO-based glycogen synthase inhibitor complimentary to the human target. GS-PPMO showed reasonable activity at reducing Gys1 mRNA, protein and activity in skeletal muscle including diaphragm, but was less effective in the heart. In contrast, rhGAA is more effective at clearing glycogen from the heart than from skeletal muscle, suggesting that the merits of the two treatment approaches are complimentary and potentially useful in some combination paradigm. More testing is needed to determine the durability of GS-PPMO efficacy and whether it is possible to employ a longer interval between administrations.

## Materials and Methods

*Design of phosphorodiamidate morpholino oligomers.* PMOs were obtained from Gene Tools, Philomath, OR. PMO sequences were designed to hybridize to Gys1 mRNA so as to invoke either exon skipping or translation inhibition as described by Morcos.^24^ The sequences designed to skip exons in Gys1 mRNA are as follows:

PMO 1 (5′-TCAGGGTTGTGGACTCAATCATGCC-3′) targeted the intronic sequence proximal to the splice acceptor site of intron 7;

PMO 2 (5′-AAGGACCAGGGTAAGACTAGGGACT-3′) targeted the intronic sequence proximal to the splice acceptor site of intron 4;

PMO 3 (5′-GTCCTGGACAAGGATTGCTGACCAT-3′) targeted the exon–intron boundary of exon 8;

PMO 4 (5′-CTGCTTCCTTGTCTACATTGAACTG-3′) targeted the intron–exon boundary of exon 5;

PMO 5 (5′-ATACCCGGCCCAGGTACTTCCAATC-3′) targeted the exon–intron boundary of exon 14;

PMO 6 (5′-CTGGACAAGGATTGCTGACCATAGT-3′), similar to PMO 3 also targeted the exon–intron boundary of exon 8;

PMO 7 (5′-AATTCATCCTCCCAGTCTTCCAATC-3′) was designed to inhibit translation initiation by targeting a sequence 3′ to the initiation codon of Gys1;

PMO 8 (5′-TCCCACCGAGCAGGCCTTACTCTGA-3′) targeted the exon–intron boundary of exon 7;

PMO 9 (5′-GACCACAGCTCAGACCCTACCTGGT-3′) targeted the exon–intron boundary of exon 5;

PMO 10 (5′-TCACTGTCTGGCTCACATACCCATA-3′) targeted the exon–intron boundary of exon 6.

*Conjugation of a cell-penetrating peptide to a morpholino oligonucleotide.* Conjugation of PMO to a cell-penetrating peptide (to generate GS-PPMO) was conducted as previously described^23^ with the following modifications. The PMO, with the sequence 5′-TCACTGTCTGGCTCACATACCCATA-3′ (referred to herein as GS-PMO) containing a 5′ primary amine modification was dissolved in dimethylsulfoxide. Peptide B (Ac(RXRRBR)2XB-OH) was preactivated in dimethylformamide containing O-(6-chlorobenzotriazol-1-yl)-N,N,N,N-tetramethyluronium hexafluorophosphate (HCTU)/diisopropylethylamine (DIEA) at a molar a ratio of 1:1:2 (Peptide:HCTU:DIEA) at room temperature (RT). Preactivated peptide was added to GS-PMO solution in molar excess of 1.2–1.5:1 (peptide:GS-PMO) and allowed to react at RT for 2 hours. The reaction was quenched with water and GS-PPMO conjugates were separated from unbound GS-PMO by chromatography over a carboxymethyl sepharose column (GE Healthcare, Piscataway, NJ) and the bound conjugates eluted with a buffer containing 2 mol/l guanidine-HCl, 1 mol/l NaCl, pH 7.5, and 20% acetonitrile. The eluate was dialyzed against several buffer exchanges of 0.1 mmol/l NH_4_HCO_3_ in a dialysis cassette with molecular weight cut-off of 3,000 Da. The dialyzed GS-PPMO was quantified by spectrophotometry in 0.1N HCl at 265 nm, frozen, and lyophilized.

*Animal studies.* Animal experiments were conducted in accordance with the Guide for the Care and Use of Laboratory Animals (US Department of Health and Human Services, NIH Publication No. 86-23) and by Genzyme's IACUC committee.

*Intramuscular injection of PMO for screening.* Six-week-old C57BL/6 male mice (Jackson Laboratories, Bar Harbor, ME) were anesthetized with isoflurane and the tibialis anterior (TA) muscle was injected as previously described.^25^ TA muscles were injected with 12 µl of 0.4U/μl bovine hyaluronidase (Sigma Aldrich, St Louis, MO) 2 hours before PMO injection and electroporation. One TA was injected with 20 µg (1 µg /µl) of various PMOs (**Table 1**) and the contralateral TA with 20 µl phosphate-buffered saline. Immediately following injection, the muscle was electroporated using the parameters of 100 V/cm, 10 pulses at 1 Hz, and 20 ms duration per pulse. Mice were euthanized 2 weeks after electroporation and TA muscle collected and snap frozen until analysis.

*Intravenous administration of GS-PPMO.* Six-week-old male and female GAA^−/−^ (Charles River Laboratories, Wilmington, MA) and C57BL/6 mice (Jackson Laboratories) were employed to evaluate the efficacy of substrate inhibition using peptide-linked morpholinos. Tissues were collected from a cohort of WT and GAA^−/−^ animals at the start of the studies to serve as a baseline reference for the glycogen accumulation assay (both groups *n* = 10; five females/five males). C57Bl/6 served as a genetic control for the duration of the experiment (*n* = 10; five females/five males). GS-PPMO was dissolved in phosphate-buffered saline and administered at 15 or 30 mg/kg bodyweight by tail vein injection once every 2 weeks for a total of 12 weeks (15 mg/kg, *n* = 10; five females/five males and 30 mg/kg *n* = 9; five females/four males). The positive control, rhGAA, was reconstituted in a buffer consisting of 25 mmol/l sodium phosphate pH 6.2, 2% mannitol, and 0.005% polysorbate 80 (Sigma Aldrich) and 20 mg/kg administered by tail vein injection once every 2 weeks for 12 weeks (buffer, *n* = 10; rhGAA, *n* = 10; both five females/five males). To minimize the potential for a hypersensitivity reaction to rhGAA, mice were intraperitoneally pretreated with 5 mg/kg diphenhydramine starting at the third dose of rhGAA. Two weeks after the final dose, the mice were euthanized, tissues collected and snap frozen in liquid nitrogen for *in vitro* analyses or fixed in 10% neutral buffered formalin for histological analysis.

*RNA analysis.* Total RNA was isolated from frozen tissues using a commercial kit with optional DNA digestion (Qiagen, Valencia, CA). RT-PCR was conducted using the SuperScript III One-Step RT-PCR System with Platinum Taq DNA Polymerase (Life Technologies, Grand Island, NY) and custom primers (Invitrogen, Grand island, NY) used for cDNA synthesis and PCR amplification. Primer sequences used were: Gys1 forward, 5′-CTGGCGCTGTGGACTTCTA-3′; Gys1 reverse, 5′-ACACTGGTGGGAAAGACTGC-3′; Gys2 forward, 5′-CCAGCTTGACAAGTTCGACA-3′; Gys2 reverse, 5′-AAACACCCCAAGGTGACAAC-3′; β-actin forward, 5′-AGCCATGTACGTAGCCATCC-3′; and β-actin reverse, 5′-CTCTCAGCTGTGGTGGTGAA-3′. RT-PCR products (following 25 cycles) were separated on 2% agarose gels containing ethidium bromide and scanned using the ChemiGenius2 bio-imaging system (Syngene, Frederick, MD). Band intensities were quantified using Image J software (NIH, Bethesda, MD). Levels of Gys1 and Gys2 mRNA were normalized β-actin levels.

*Preparation of tissue homogenates.* Frozen tissues were reconstituted in 6 volumes (vol/wt) of homogenization buffer (20 mmol/l Tris/HCl, pH 7.5, 150 mmol/l NaCl, 25 mmol/l β-glycerophosphate, 20 mmol/l sodium fluoride, 1 mmol/l sodium orthovanadate, 2 mmol/l sodium pyrophosphate, 2 mmol/l ethylenediaminetetraacetic acid and Roche complete protease inhibitor cocktail (Roche Applied Science, Indianapolis, IN) and then homogenized using a Tissue Lyser II (Qiagen) and frozen overnight at −80 °C. Lysates were thawed then centrifuged at 1,200 × g for 15 minutes at 4 °C and the supernatants collected and stored at −80 °C. Protein concentrations were determined using the Micro BCA kit as described by the manufacturer (Pierce, Rockford, IL).

*Western blot analysis of tissue lysate.* Tissue homogenates (50–100 µg protein) were boiled in sample buffer (Invitrogen) containing dithiothreitol and applied to a gradient (4–15%) Tris/HCl-polyacrylamide gel (BioRad, Hercules, CA). Following electrophoresis, the separated proteins were transferred onto nitrocellulose using a dry blot apparatus (iBlot, Invitrogen). The nitrocellulose blots were first blocked overnight with 3% nonfat milk and the appropriate antibody (Cell Signalling, Danvers, MA) then added to a final concentration of 0.02–0.08 ng/ml and incubated for 1 hour at RT. Blots were then incubated with a horse radish peroxidase–conjugated secondary antibody (Jackson Immuno research, West Grove, PA) for 1 hour at room temperature and treated with an ECL substrate detection kit as described by the manufacturer (Pierce). Protein band intensities were quantified using Image J software (NIH). Levels of glycogen synthase 1 and 2 protein were normalized to glyceraldehyde 3-phosphate dehydrogenase levels.

*Glycogen synthase activity assay.* Glycogen synthase activity in tissue lysates was measured using a gel filtration radioactivity assay as described previously.^26^ A 60 µl reaction solution consisting of 10 µg of protein lysate (2 ng/µl), 4% glycogen, 30 mmol/l UDP-glucose, 4.5 mmol/l glucose-6-phosphate (Sigma Aldrich), homogenization buffer (described above) and labeled uridine diphosphate glucose [Glucose-^14^C-U] (Perkin Elmer, Waltham, MA) was incubated in a 37 °C water bath for 1 hour, the reaction was stopped with 0.6N perchloric acid, and 50 µl of the reaction was loaded onto a quick spin (G-50) sephadex columns (Roche, Indianopolis, IN) and centrifuged at 1,000 × g for 4 minutes. The eluted radiation was added to 4 ml Ultima Gold LSC cocktail (Perkin Elmer) and radiation was measured using a scintillation counter (Beckman Coulter, Indianapolis, IN). Enzyme activity was calculated by determining the amount of UDP-[U-^14^C]-glucose incorporated into glycogen per minute per milligram of protein

*Measurement of tissue glycogen levels.* Tissue glycogen levels were determined as previously described^15^ using an Amplex Red Glucose/Glucose Oxidase Assay Kit (Molecular Probes, Eugene, OR) per the manufacturer's instructions. Fluorescence was detected and analyzed using a Spectra Max M3 micro-plate reader, 530 nm excitation and 590 nm emission, with Softmax Pro5 acquisition and analysis software (Molecular Devices, Sunnyvale, CA). Rabbit liver glycogen (Sigma-Aldrich) was used to construct the standard curve. Glycogen levels were determined by subtracting the glucose levels in the undigested samples from those in the digested samples.

*Serum chemistry.* Whole blood was collected in serum separator tubes (BD, Franklin Lakes, NJ) from the retro-orbital plexus of anesthetized mice 1 hour after the final dose of GS-PPMO. Blood was allowed to clot for thirty minutes and then centrifuged at 1,300 × g for 15 minutes. Serum was dispensed as aliquots and frozen at −20 °C until analysis. *In vitro* diagnostic quantitative determination of chemistry analytes in serum was performed by spectrophotometric methods at 37 °C using the VetACE clinical chemistry system (Alfa Wassermann, West Caldwell, NJ) in accordance to the manufacturer's specifications.

*Histology.* Kidney and liver were collected from mice following euthanasia, fixed for up to 72 hours in 10% neutral buffered formalin and processed for paraffin embedding.^27^ Serial 5 µm-thick sections were generated and stained with hemotoxylin and eosin solution (Richard Allen Scientific, Kalamazoo, MI). A board certified veterinary pathologist, blinded-to the treatments, evaluated the slides for qualitative analysis.

*Statistical analysis.* Data are expressed as mean ± SEM. Data analysis was performed with SigmaStat Software (Systat Software, San Jose, CA) using one-way analysis of variance and Tukey *post hoc* analysis. A probability value of *P* < 0.05 was considered to be statistically significant.

## Figures and Tables

**Table 1:**
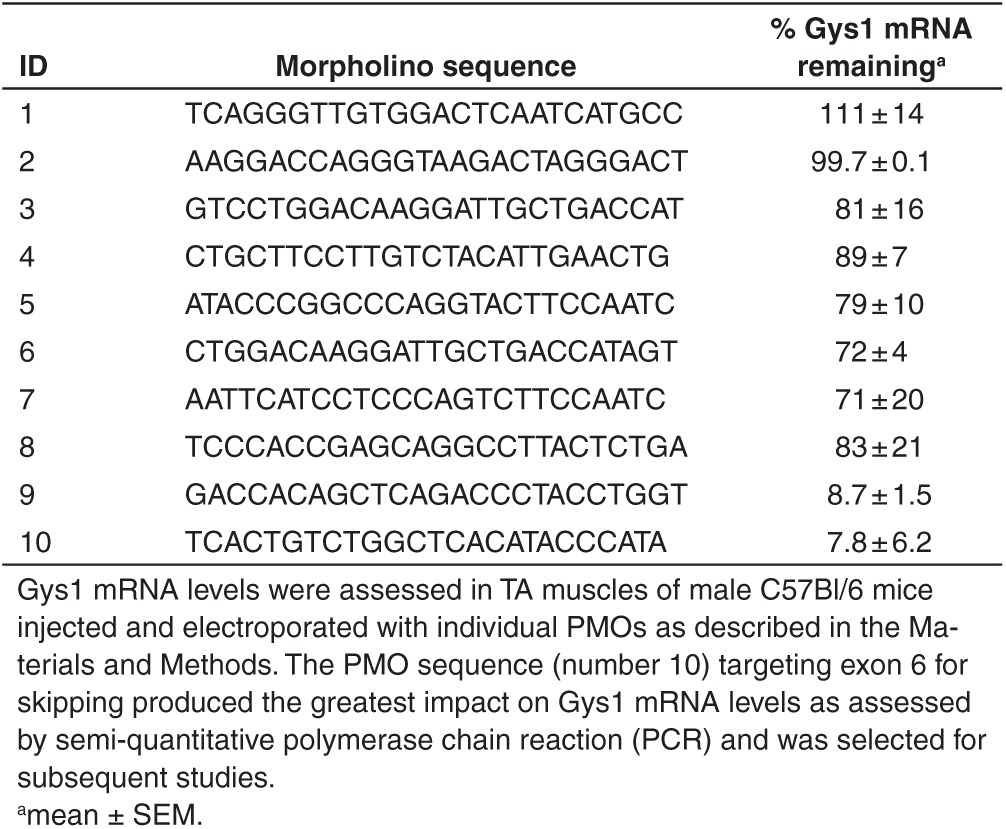
Identification of active phosphorodiamidate morpholino oligomer (PMO) sequences designed to target Gys1
